# Reviews and Notices

**Published:** 1886-11-27

**Authors:** 


### Reviews and Notices.

### AMBULANCE LECTURES.*

Somebody (was it Charles Reade?) wrote a novel, of which the title was *Put Yourself in His Place.* That title—not the book—we commend to Dr. Martin's studious consideration. How is it that men who speak and write will not take the trouble to realise what it is their hearers and readers do not, and what it is they do, want to know? We are impelled to make these remarks by the complete failure of Dr. Martin to realise the difficulties of an outsider who comes to the study of elementary anatomy and surgery. Dr. Martin's book contains half a dozen ambulance lectures. Ambulance lectures are just now very popular institutions, and they may be made both pleasant and useful. But then, seeing that they deal with subjects of which the uninitiated know absolutely nothing, they must be prepared with adequate skill and care, and with due regard for the difficulties of the learner. Dr. Martin's lectures entirely fail in these very important particulars. Here is a case in point, selected from the very first lecture. The author says (p. 11): "The cause of the circulation is the difference of pressure which exists between the blood in the aorta and pulmonary artery on the one hand, and the two venæ cavæ and the four pulmonary veins on the other." Now what possible meaning can any uninstructed person extract from a sentence like that? Of course, if the way had been prepared by an explanation of the structure and arrangement of the heart and vessels, or if such an explanation were given immediately thereafter, some degree of sense might be got out of it. But will it be believed that there is not a word of explanation or description either before or after which in any degree helps the reader who really desires to learn? In the passage quoted, the following five highly technical terms are employed: "pressure," "aorta," "pulmonary artery," "venæ cavæ," "pulmonary veins." Will it be credited, we again ask, that in a book written entirely for outsiders, and offered as a model of what ambulance lectures should be, not one word is given explanatory of the meaning of any of these terms in the whole so-called description of the circulation? What kind of ideas does Dr. Martin suppose his hearers got into their heads from his description? He might just as well have told a class of infants in arms that "the square of the hypothenuse of a right-angled triangle is equal to the squares of the other two sides," or any other equally true, but equally, to them, incomprehensible thing. Of course, there are diagrams and references—but what is the use of a diagram without a description? You might as well give a man a painted sirloin and tell him to make his dinner off it. There are other mistakes in plenty in the book, which are evidently the result of carelessness or want of skill; for example, we are told on page 6 that "the thorax is a bony cavity . . . . It is formed by *twelve* ribs, etc." Dr. Martin, of course, means *twelve on each side*—that is to say, *twenty-four ribs;* but how does he suppose his readers are to know he means *twenty-four*, when he says *twelve?* On page 18 is given a description of the brain,

* *Ambulance Lectures*, by J. H. Martin, M.D. London: J. and A. Churchill.

which is so brief, so incomprehensible, and so useless, that we call special attention to it as a perfect model of what a description should *not* be. "The brain," says Dr. Martin, "is contained within the cavity of the skull, and consists of two portions—the cerebrum or large brain, and the cerebellum or small brain. In the former, the intelligence, the emotions, and the will reside; in the latter, *the motions of the body*, the regulation of equilibrium, and combined movement." How can "the motions of the body reside" in the brain? No doubt Dr. Martin means that the *cause* of motion is, in a sense, in the brain and cord: but if that be what he means, why does he not say so? And if he gives any description of the brain at all, why does he not give one that shall at least convey some little glimmer of meaning to his bewildered audience?

The book is small, and it may appear that we have dealt with it at disproportionate length; but we are anxious that the public who attend or read ambulance lectures should not be led into a hopeless maze of semi-scientific word-spinning, and from their experience come to the conclusion that science has nothing practical or useful to tell them on such questions. Science has very much to tell them, and when she finds an able expositor, she can make herself as delightful as she is instructive and profitable. When Dr. Martin, or any other teacher, undertakes again to inform and guide the public, let him take a bright little boy of ten into his study, and go over the subjects of his lectures beforehand with him by way of question and answer,—then, perhaps, he will both speak and write so as to be "understanded of the people."

